# Optimization of Exergy Output Rate in a Supercritical CO_2_ Brayton Cogeneration System

**DOI:** 10.3390/e27101078

**Published:** 2025-10-18

**Authors:** Jiachi Shan, Shaojun Xia, Qinglong Jin

**Affiliations:** 1 College of Power Engineering, Naval University of Engineering, Wuhan 430033, China; m24380703@nue.edu.cn; 2 Department of Electromechanical Engineering, Faculty of Science and Technology, University of Macau Taipa, Macau, China; yc47440@um.edu.mo

**Keywords:** supercritical CO_2_ Brayton cycle, finite-time thermodynamics, exergy output rate, cogeneration

## Abstract

To address low energy utilization efficiency and severe exergy destruction from direct discharge of high-temperature turbine exhaust, this study proposes a supercritical CO_2_ Brayton cogeneration system with a series-connected hot water heat exchanger for stepwise waste heat recovery. Based on finite-time thermodynamics, a physical model that provides a more realistic framework by incorporating finite temperature difference heat transfer, irreversible compression, and expansion losses is established. Aiming to maximize exergy output rate under the constraint of fixed total thermal conductance, the decision variables, including working fluid mass flow rate, pressure ratio, and thermal conductance distribution ratio, are optimized. Optimization yields a 16.06% increase in exergy output rate compared with the baseline design. The optimal parameter combination is a mass flow rate of 79 kg/s and a pressure ratio of 5.64, with thermal conductance allocation increased for the regenerator and cooler, while decreased for the heater. The obtained results could provide theoretical guidance for enhancing energy efficiency and sustainability in S-CO_2_ cogeneration systems, with potential applications in industrial waste heat recovery and power generation.

## 1. Introduction

### 1.1. S-CO_2_ Background and Motivation

Gas turbines, valued for their compact size and light weight [1], are widely deployed in modern marine propulsion. However, their exhaust gases remain at very high temperatures, and direct discharge leads to significant energy loss. Efficient recovery of this waste heat is therefore essential. Supercritical CO_2_ (S-CO_2_), with its high density in the supercritical state and excellent heat transfer properties [2,3,4], offers an attractive working fluid for such systems, enabling smaller compressors and turbines. In recent years, an increasing number of countries and research teams have invested resources in the development of S-CO_2_ Brayton cycle energy conversion technology.

Early studies of S-CO_2_ Brayton systems applied classical thermodynamic analysis to assess efficiency and parameter sensitivities. For example, Yildiz et al. [5] explored recompression cycles for hydrogen production, while Zhao et al. [6] used genetic algorithms to optimize single- vs. multi-stage compression. Other works [7] demonstrated the potential of integrating S-CO_2_ cycles with marine gas turbines for waste heat recovery, achieving significant gains in thermal and exergy efficiency. These studies established the foundation for S-CO_2_ cycle applications but generally assumed idealized, internally reversible processes.

### 1.2. Limitations of Classical Thermodynamics and the Development of FTT

Classical thermodynamics assumes infinite process duration and neglects finite temperature difference heat transfer irreversibility, limiting its predictive power for real systems. Finite time thermodynamics (FTT), developed from the 1970s onward [8,9,10,11,12,13,14,15], addressed these limitations by incorporating finite temperature differences, irreversibility, and time constraints into system models. Bejan’s Entropy Generation Minimization [16,17,18,19,20,21] and subsequent studies [22,23,24,25,26,27,28] demonstrated how FTT could optimize practical cycles, from Brayton systems to compressors, by balancing efficiency and power output. Today, FTT has become a critical framework linking theoretical thermodynamics with engineering design. Since the beginning of the 21st century, FTT has demonstrated unprecedented potential across various fields, becoming a core bridge connecting fundamental disciplines and engineering practices. As of September 2025, incomplete statistics show that 26,000 related publications have been released.

### 1.3. Recent Research on S-CO_2_ Brayton Cycles

More recent research has applied FTT to recuperated S-CO_2_ Brayton cycles and combined heat and power (CHP) configurations. Studies have investigated power output optimization [29,30,31], cascade waste heat utilization [32], and performance improvements via pressure ratio control [33]. Jin et al. [34,35,36] highlighted the characteristic loop-shaped relation between pressure ratio, net power, and efficiency in recuperated cycles, while others [37,38,39] examined integration with CHP, nuclear, and solar systems. These works confirm the potential of S-CO_2_ cycles for high-efficiency waste heat recovery, but they also reveal persistent gaps: most analyses focus on efficiency or net power, while the mechanisms of irreversibility and their influence on exergy-based performance remain less explored.

### 1.4. Research Gap and Contributions of This Study

Although significant progress has been made in the modeling and optimization of recuperated, preheating, and recompression S-CO_2_ cycles [34,35,36,40,41], most existing studies employ parallel configurations to optimize the “front-end” (i.e., heat source side) of the cycle, with the core objective typically being the maximization of power generation efficiency or net power. Deviating from this mainstream paradigm, this study proposes a series configuration by integrating a series-connected hot water heat exchanger, thereby shifting the optimization perspective to the “back-end” or “user-end” of the cycle. The fundamental purpose of this design is to achieve cascade utilization of energy, i.e., effectively recovering the residual thermal exergy of the working fluid for heating after power generation, thus constructing a combined heat and power system that genuinely addresses end-user demands.

Despite these advances, there is still insufficient theoretical support for CHP systems recovering low-grade waste heat, particularly regarding how irreversibilities shape exergy performance. To address this gap, the present study develops an exergy analysis model for a recuperated S-CO_2_ Brayton CHP system, explicitly considering finite time heat transfer, irreversible compression, and expansion losses. The optimization objective is the maximization of exergy output rate. A neural-network-assisted strategy is employed to optimize decision variables, including mass flow rate, cycle pressure ratio, and thermal conductance distribution across heat exchangers. This step-wise optimization framework provides new insights into exergy-based design of S-CO_2_ CHP systems.

## 2. Physical Model

**Figure 1:**
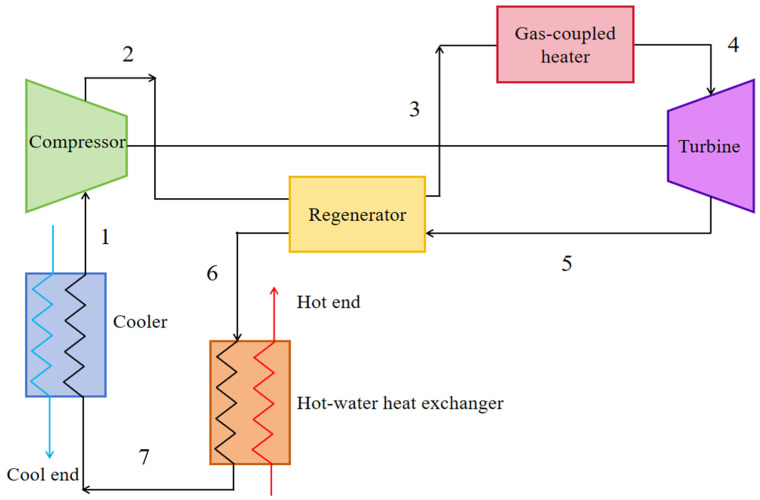
System diagram of a regenerated S-CO_2_ Brayton cycle cogeneration plant.

**Figure 2:**
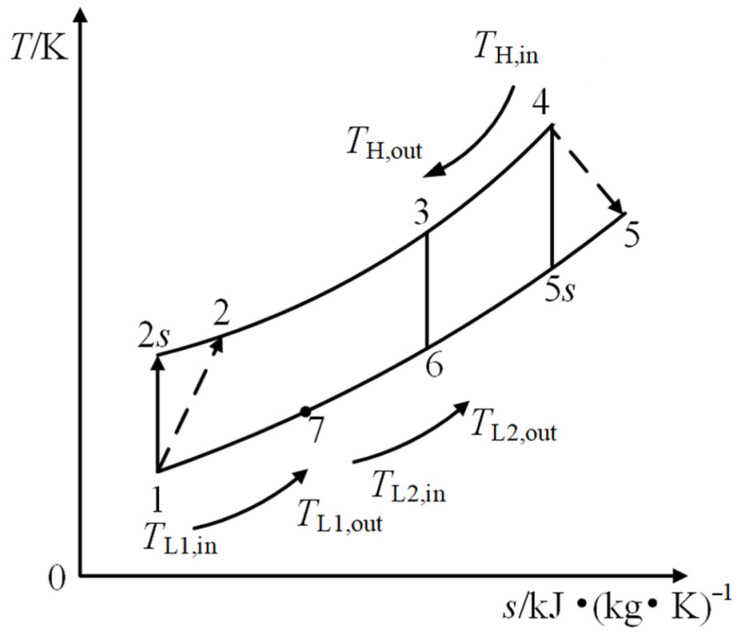
The *T-s* diagram of regenerated S-CO_2_ Brayton cycle cogeneration plant.

Figure 1 shows the system diagram of a regenerated S-CO_2_ Brayton cycle cogeneration plant. The proposed recuperated S-CO_2_ Brayton CHP system comprises a compressor, turbine, recuperator, gas-coupled heater, cooler, and a hot-water heat exchanger for cogeneration. Figure 2 shows the T–s diagram of the corresponding thermodynamic cycle. As shown in Figure 2, process 1–2 represents the ideal isentropic compression process in the compressor, while process 1–2 represents the actual irreversible adiabatic compression process. Processes 2–3 and 3–4 correspond to the constant-pressure heat absorption processes in the recuperator and the heater, respectively. Process 4–5 represents the actual irreversible adiabatic expansion process in the turbine, whereas process 4–5 denotes the ideal isentropic expansion process. Process 5–6 is the constant-pressure heat rejection process in the recuperator. Process 6–7 involves the working fluid transferring heat to the low-temperature heat sink in the water heater. Finally, process 7–1 describes the cooling of the working fluid in the cooler to reach the compressor inlet conditions before it re-enters the compressor for the next compression cycle.

Based on the first law of thermodynamics, the turbine isentropic efficiency ($\eta_{t}$) is defined as the ratio of the actual enthalpy drop of S-CO_2_ to the ideal isentropic enthalpy drop during expansion, i.e., Equation (1). Similarly, the compressor’s entropic efficiency ($\eta_{c}$) is defined as the ratio of the ideal isentropic enthalpy rise to the actual enthalpy rise during compression, i.e., Equation (2). These definitions reflect irreversibility losses in turbomachinery. They are, respectively, expressed as:

(1) $$\eta_{t}=\left(h_{4}-h_{5}\right)/\left(h_{4}-h_{5s}\right)$$

(2) $$\eta_{c}=\left(h_{2s}-h_{1}\right)/\left(h_{2}-h_{1}\right)$$

Based on the thermodynamic properties of the working fluid, heat transfer between the working fluid and heat sources, heat exchanger theory, and the properties of the heat sources [34,35,36,37,40,41,42,43,44], the values of $Q_{H}$, $Q_{L1}$, $Q_{L2}$ and ${\;Q}_{R}$, for the system are, respectively, given by [45,46,47,48,49]:

(3) $$Q_{H}={\mathrm{UA}}_{H}\cdot \frac{\left(T_{H,\mathrm{in}}-T_{4}\right)-\left(T_{H,\mathrm{out}}-T_{3}\right)}{\ln\left[\left(T_{H,\mathrm{in}}-T_{4}\right)-\left(T_{H,\mathrm{out}}-T_{3}\right)\right]}=c_{p,H}\cdot m_{H}\left(T_{H,\mathrm{in}}-T_{H,\mathrm{out}}\right)=m_{\mathrm{wf}}\left(h_{4}-h_{3}\right)$$

(4) $$Q_{L1}={\mathrm{UA}}_{L1}\cdot \frac{\left(T_{7}-T_{L1,\mathrm{out}}\right)-\left(T_{1}-T_{L1,\mathrm{in}}\right)}{\ln\left[\left(T_{7}-T_{L1,\mathrm{out}}\right)-\left(T_{1}-T_{L1,\mathrm{in}}\right)\right]}=c_{p,L}\cdot m_{L1}\left(T_{L1,\mathrm{out}}-T_{L1,\mathrm{in}}\right)=m_{\mathrm{wf}}\left(h_{7}-h_{1}\right)$$

(5) $$Q_{L2}={\mathrm{UA}}_{L2}\cdot \frac{\left(T_{6}-T_{L2,\mathrm{out}}\right)-\left(T_{7}-T_{L2,\mathrm{in}}\right)}{\ln\left[\left(T_{6}-T_{L2,\mathrm{out}}\right)-\left(T_{7}-T_{L2,\mathrm{in}}\right)\right]}=c_{p,L2}\cdot m_{L2}\left(T_{L2,\mathrm{out}}-T_{L2,\mathrm{in}}\right)=m_{\mathrm{wf}}\left(h_{6}-h_{7}\right)$$

(6) $$Q_{R}={\mathrm{UA}}_{R}\cdot \frac{\left(T_{5}-T_{3}\right)-\left(T_{6}-T_{2}\right)}{ln\left[\left(T_{5}-T_{3}\right)-\left(T_{6}-T_{2}\right)\right]}=m_{\mathrm{wf}}\left(h_{3}-h_{2}\right)=m_{\mathrm{wf}}\left(h_{5}-h_{6}\right)$$

In these equations, $Q_{H}$ is the heat absorption rate (kW) from the high-temperature source in the heater, $Q_{L1}$ is the heat rejection rate in the cooler (kW) to the low-temperature sink in the cooler, $Q_{L2}$ is the heat rejection rate in the water heater (kW) to the water in the water heater, and $Q_{R}$ is the heat transfer rate in the recuperator (kW) within the regenerator. $T_{L1,\mathrm{in}}$ is the inlet temperature of the cooling water in the cooler (K), $T_{L1,\mathrm{out}}$ is the outlet temperature of the cooling water in the cooler (K), $T_{L2,\mathrm{in}}$ is the inlet temperature of the cooling water in the water heater (K), and $T_{L2,\mathrm{out}}$ is the outlet temperature of the cooling water in the water heater (K). $T_{6}$ and $T_{7}$ are the inlet and outlet temperatures, respectively, of the S-CO_2_ working fluid in the water heater (K). $c_{p,L}$ is the specific heat capacity at constant pressure of the cooling water in the cooler (kJ/(kg·K)). $m_{L1}$ is the mass flow rate of the cooling water in the cooler (kg/s) and $m_{L2}$ is the mass flow rate of the cooling water in the water heater (kg/s).

In Equations (3)–(6), the log mean temperature difference (LMTD) formulation is derived based on constant fluid specific heat capacities (*c*_p_). It is important to clarify the hierarchical strategy employed in our modeling to balance accuracy and computational efficiency. For the S-CO_2_ working fluid, its properties at all thermodynamic state points (e.g., enthalpy, entropy) are dynamically calculated by invoking the REFPROP database via MATLAB R2024b, thereby fully accounting for its significant variation in specific heat capacity with temperature and pressure. This ensures accuracy in the energy balance calculations. For the external fluids (heat source and cold source), within the LMTD terms, their specific heat capacities are treated as constants. Here, *c*_p,H_ and *c*_p,L_ are defined as representative values evaluated at the average temperature of the respective fluid across the inlet and outlet of the corresponding heat exchanger. The purpose is to establish a stable and solvable optimization model capable of effectively revealing system performance trends and optimal parameter ranges.

The total thermal conductance for the cycle is:

(7) $${\mathrm{UA}}_{T}\;=\;{\mathrm{UA}}_{H}+{\mathrm{UA}}_{R}+{\mathrm{UA}}_{L1}+{\mathrm{UA}}_{L2}$$

(8) $$\psi_{i}=\frac{UA_{i}}{UA_{T}}$$

where the thermal conductance allocation ratio, $\psi_{i}$, for each heat exchanger is defined as the ratio of its thermal conductance (*UA_i_*) to the total thermal conductance of the system (*UA*_T_), consistent with the definition provided in Na et al. [41]. ${\mathrm{UA}}_{T}$ is the total thermal conductance (kW/K), ${\mathrm{UA}}_{H}$ is the thermal conductance of the heater (kW/K), ${\mathrm{UA}}_{R}$ is the thermal conductance of the recuperator (kW/K), ${\mathrm{UA}}_{L1}$ is the thermal conductance of the cooler (kW/K), and ${\mathrm{UA}}_{L2}$ is the thermal conductance of the water heater (kW/K). The total thermal conductance is related to the cycle system design and the working fluid. In practical engineering applications, the total thermal conductance of the heat exchangers is constrained by the overall size of the system. Considering these practical engineering limitations, the total thermal conductance is set as a constant value. The thermal conductance allocation ratio, *Ψ*, defined as the ratio of an individual heat exchanger’s thermal conductance to the total thermal conductance, characterizes the relative heat transfer capacity of each heat exchanger. Thus, the thermal conductance allocation ratio for the heater is $\psi_{H}={\mathrm{UA}}_{H}/{\mathrm{UA}}_{T}$, for the recuperator is $\psi_{R}={\mathrm{UA}}_{R}/{\mathrm{UA}}_{T}$, for the cooler is $\psi_{L1}={\mathrm{UA}}_{L1}/{\mathrm{UA}}_{T}$, and for the water heater is $\psi_{L2}={\mathrm{UA}}_{L2}/{\mathrm{UA}}_{T}$. By optimizing the thermal conductance allocation among the heat exchangers, the exergy output rate of the system can be maximized. Because the total thermal conductance is constrained by heat exchanger volume and system compactness, optimization involves redistributing conductance among components. The allocation ratios $\psi_{H}$, $\psi_{R}$, $\psi_{L1}$, and $\psi_{L2}$ quantify this distribution, and must satisfy Equation (9). The following relationship exists:

(9) $$\psi_{H}+\psi_{R}+\psi_{L1}+\psi_{L2}=1$$

The total heat release rate from the exhaust gas to the system is expressed by Equation (10) as follows:

(10) $$Q_{\mathrm{hg}}=m_{H}\left(h_{H,\mathrm{in}}-h_{H,\mathrm{out}}\right)$$

In Equation (10), $Q_{\mathrm{hg}}$ represents the total heat release rate from the exhaust gas to the system.

The exergy output rate of the cogeneration system considers both the quantity of the output energy and its quality (which affects the upper limit of the exergy output rate). Based on the second law of thermodynamics and incorporating an exergy correction for low-grade thermal energy that accounts for the environmental temperature (*T*_0_), the total exergy output rate is defined as the sum of the net power output exergy and the exergy associated with the heat supplied to the user. It is defined by the following expression:

(11) $$P_{\mathrm{out}}=Q_{\mathrm{hg}}-Q_{L1}-Q_{L2}$$

(12) $$P_{\mathrm{out}}=P_{\mathrm{net}}=W_{t}-W_{c}=Q_{\mathrm{hg}}-Q_{L1}-Q_{L2}$$

(13) $$E_{K}=Q_{L2}-T_{0}\cdot m_{L2}\cdot c_{p,L}\cdot \ln\left(\frac{T_{L2,\mathrm{out}}}{T_{L2,\mathrm{in}}}\right)$$

(14) $$E_{\mathrm{out}}=P_{\mathrm{out}}+E_{K}$$

In the above equations, $P_{\mathrm{out}}$ represents the net power output of the system. Applying the first law of thermodynamics to the entire system, the net power output must also equal the net heat transfer rate: the total heat input from the hot gas ($Q_{\mathrm{hg}}$) minus the total heat rejected to the cold reservoirs ($Q_{L1}+Q_{L2}$). $E_{K}$ is the exergy contribution of useful low-grade heat delivered to users, and $E_{\mathrm{out}}$ is the total exergy output rate of the system.

## 3. Results and Discussion

### 3.1. Model Validation

Currently, the theoretical research on the thermoelectric combined S-CO_2_ Brayton cycle lacks experimental references, making validation relatively challenging. As indicated by the cycle process, it can be simplified as a recuperative S-CO_2_ cycle, with the main difference being the addition of a hot water heat exchanger. To facilitate model validation, the thermal conductance distribution ratio $\psi_{L2}$ of the hot water heat exchanger is set to 0. Reference [50] utilized CFD 2017 software for the analysis and optimization of the recuperative cycle, and simulation data for the recuperative Brayton cycle were obtained based on Gate Cycle 6.1.4 software, supported by relevant experiments. In this study, data from Reference [50] are used for comparative calculation via MATLAB, as shown in Table 1. Since the reference did not analyze exergy efficiency but focused primarily on net power output, the net power results of the present cycle are compared with those in the literature. As shown in Table 2, the maximum discrepancy in state points between the thermoelectric combined cycle and the recuperative cycle in the literature is 3.57%, and the net power discrepancy is 2.50%. The deviations observed at state points (e.g., T_2_, T_5_) primarily stem from the fact that the reference model in Reference [50] accounts for pressure drops in heat exchangers and flow processes, whereas such internal component pressure losses are not yet incorporated in the present model. This leads to non-systematic deviations in the parameters at certain state points. Nevertheless, the calculated performance indicators show good agreement with those reported in Reference [50], demonstrating the reliability of the computational model established in this study.

To further validate the generality of the model under unequal compressor and turbine isentropic efficiencies, an additional validation case was conducted. The compressor efficiency $\eta_{c}$ was set to 0.85, and the turbine efficiency $\eta_{t}$ to 0.8, while keeping all other parameters consistent with Table 1. The comparative results are presented in Table 3. The maximum discrepancy in state points is 3.92% at *T*_6_, and the net power discrepancy is −1.25%. These deviations are comparable to those in the equal-efficiency case, confirming that the model remains reliable even when compressor and turbine efficiencies are unequal. The deviations in the unequal-efficiency case are within acceptable limits, similar to the equal-efficiency case, further verifying the model’s applicability across different efficiency settings. This confirms that the modeling approach is general and can handle variations in compressor and turbine efficiencies without loss of accuracy.

### 3.2. Exergy Output Rate Analysis of System

The exergy output rate of the cogeneration system considers both the quantity and the quality (energy grade) of the output energy. The total exergy output rate is equal to the sum of the net power exergy output and the exergy output of the heat supplied to the user. In this section, the exergy output rate $E_{\mathrm{out}}$, derived from Equation (12), is taken as the objective function. The system performance is first analyzed and then optimized to maximize the exergy output rate of the cogeneration system. According to Reference [41], the heat source is set as high-temperature exhaust gas from a gas turbine, with its composition consisting of 78.12% nitrogen, 20.96% oxygen, and 0.92% argon. Environmental cold water is used as the low-temperature cold source. The constant-pressure specific heat capacities ($c_{p}$) of the heat and cold sources are dynamically calculated by invoking the REFPROP property database via the MATLAB platform [34,35,36]. The complete property parameters and boundary conditions are listed as the initial design parameters in Table 4.

**Figure 3:**
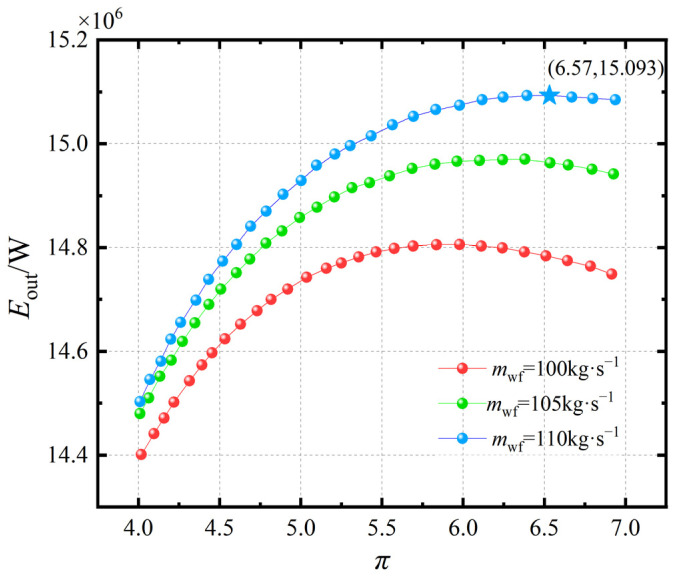
Effect of $m_{\mathrm{wf}}$ on the $E_{\mathrm{out}}$-π relationship.

Figure 3 illustrates the variation in the system’s exergy output rate under pressure ratios π ranging from 4 to 7 and working fluid mass flow rates $m_{\mathrm{wf}}$ between 100 $\mathrm{kg}\cdot s^{-1}$ and 110 $\mathrm{kg}\cdot s^{-1}$. As shown in Figure 3, the exergy output rate $E_{\mathrm{out}}$ of the system initially increases and then decreases with rising pressure ratio π. For each given $m_{\mathrm{wf}}$, there exists an optimal pressure ratio that maximizes $E_{\mathrm{out}}$, and the maximum value of $E_{\mathrm{out}}$ increases as $m_{\mathrm{wf}}$ decreases. As will be revealed in the subsequent optimization, this trend occurs because the working fluid mass flow rate has not yet reached its optimal value. This is because when π is small, the system’s work capacity is relatively weak, and the increase in $E_{\mathrm{out}}$ tends to be primarily attributed to the rise in $m_{\mathrm{wf}}$. When π becomes larger, the system exhibits stronger work capability; however, due to the dominance of irreversible losses within the cycle and finite temperature difference heat transfer, $E_{\mathrm{out}}$ begins to decline with further increase in $m_{\mathrm{wf}}$ beyond a critical point. At lower pressure ratios, increasing π enhances turbine work output more than it raises compressor work, leading to higher $E_{\mathrm{out}}$. Beyond the optimum, however, irreversibilities from compression and heat transfer dominate, causing a decline. This parabolic behavior is consistent with findings by Jin et al. [34,35,36], confirming that an optimal *π* exists for maximizing Brayton cycle efficiency under finite-time constraints. Consequently, the exergy output rate only reaches a relative maximum rather than an absolute maximum.

**Figure 4:**
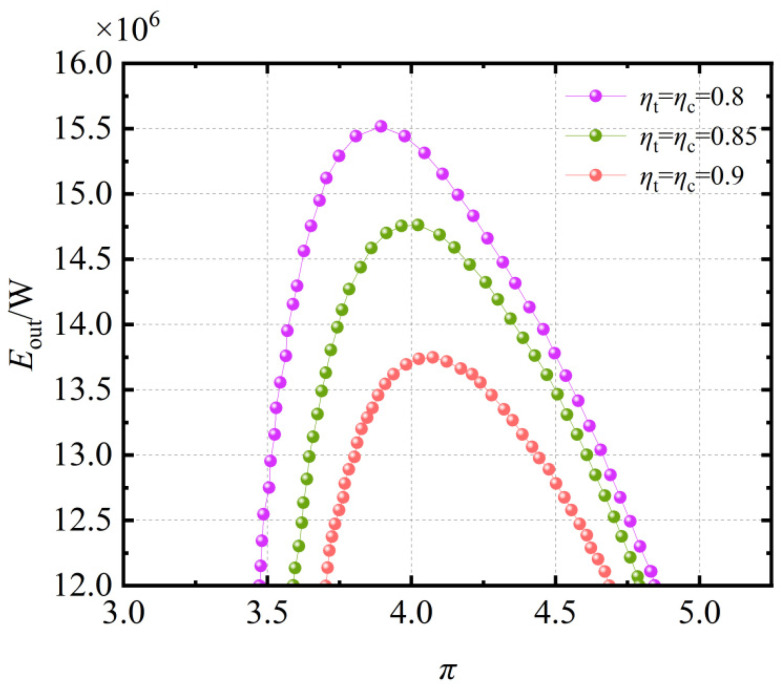
Effect of $\eta_{t}$, $\eta_{c}$ on the $E_{\mathrm{out}}$-π relationship.

Figure 4 shows the relationship between the exergy output rate $E_{\mathrm{out}}$ and the pressure ratio at different isentropic efficiency values of $\eta_{t}$ = $\eta_{c}$ = 0.8, 0.85, and 0.9. As observed in Figure 4, when $\eta_{t}{,\;\eta }_{c}$ are held constant, the exergy output rate $E_{\mathrm{out}}$ initially increases and then decreases with increasing pressure ratio π. Moreover, as $\eta_{t}$, $\eta_{c}$ increase, the exergy output rate $E_{\mathrm{out}}$ of the system gradually improves. This is because higher $\eta_{t}$, $\eta_{c}$ values reduce irreversibility losses in the system. Moreover, for each given pair of $\eta_{t}$ and $\eta_{c}$, there exists an optimal pressure ratio π that maximizes $E_{\mathrm{out}}$. The results highlight the strong sensitivity of $E_{\mathrm{out}}$ to turbomachinery performance. A reduction in $\eta_{t}$ and $\eta_{c}$ from 0.9 to 0.8 decreases the peak $E_{\mathrm{out}}$ by nearly 20%, emphasizing the importance of high-efficiency compressors and turbines in practical implementations.

**Figure 5:**
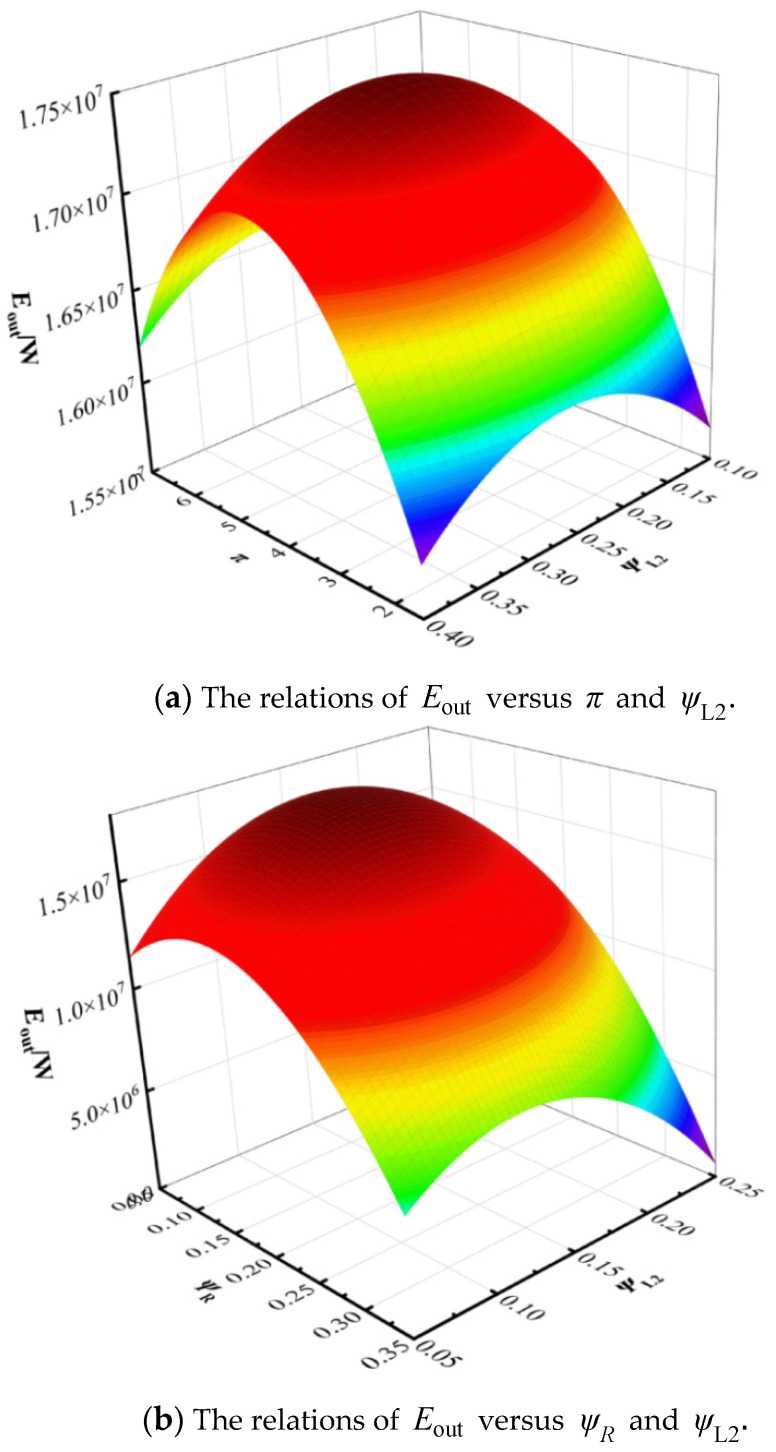
Three-dimension relationship among $E_{\mathrm{out}}$, π and $\psi_{L2}$ and three-dimension relationship among $E_{\mathrm{out}}$, $\psi_{R}$ and $\psi_{L2}$.

Figure 5a presents a three-dimensional plot showing the relationship between the exergy output rate $E_{\mathrm{out}}$, pressure ratio π, and the thermal conductance allocation ratio of the water heater $\psi_{L2}$. As observed in Figure 5, under the given operating conditions, when the pressure ratio π is held constant, increasing the thermal conductance allocation ratio of the water heater $\psi_{L2}$ leads to a reduction in the system’s exergy output rate $E_{\mathrm{out}}$. This occurs because, under the constraint of a fixed total thermal conductance, an increase in $\psi_{L2}$ results in a decrease in the allocation ratios for the other heat exchangers. Consequently, both the heat absorption and heat rejection of the cycle are reduced, leading to degraded cycle performance. The results indicate that further optimization of the cycle parameters is necessary to achieve the optimal exergy output rate of the system.

Figure 5b displays a three-dimensional representation of the exergy output rate $E_{\mathrm{out}}$ as a function of the recuperator thermal conductance allocation ratio $\psi_{R}$, which varies from 0.05 to 0.25, and the water heater thermal conductance allocation ratio $\psi_{R}$, which ranges from 0.05 to 0.35. It can be seen from Figure 5 that when the values of both $\psi_{R}$ and $\psi_{L2}$ are relatively high, the exergy output rate $E_{\mathrm{out}}$ of the cycle decreases significantly. As shown in Figure 5b, increasing $\psi_{L2}$ beyond ~0.2 reduces $E_{\mathrm{out}}$ because the additional conductance allocated to the hot-water heat exchanger comes at the expense of the heater and recuperator, weakening both heat absorption and regenerative recovery. Similarly, high $\psi_{R}$ values can also degrade performance, as excessive regeneration reduces available temperature differences for effective heat transfer. These results demonstrate the need for a balanced allocation strategy rather than simply maximizing conductance in one component.

This study aims to maximize the exergy output rate $E_{\mathrm{out}}$ as the optimization objective for cycle performance enhancement.

**Figure 6:**
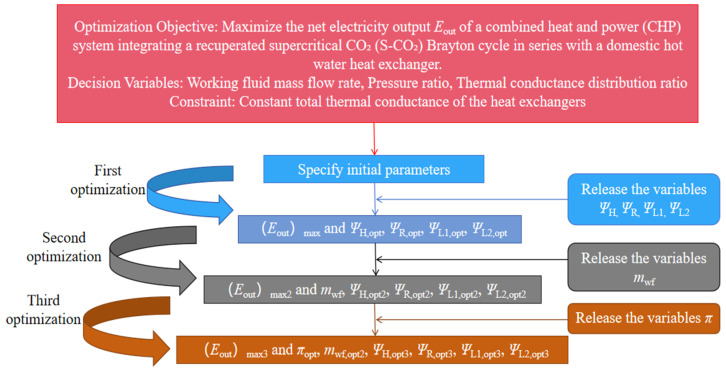
Optimization flow chart.

Figure 6 shows the optimization flow chart. The optimization was performed in three progressive stages: (i) conductance allocation optimization under fixed flow and pressure ratio, (ii) joint optimization of flow rate and conductance allocation, and (iii) full system optimization including pressure ratio. This hierarchical approach prevents premature convergence and ensures global optimality.

### 3.3. Performance Optimization

By varying the working fluid mass flow rate, thermal conductance allocation ratios, and pressure ratio, sample points of the cycle are computed. Under the constraints specified in Equation (15), a neural network-based prediction approach is employed for single-objective optimization of the cycle. This method dynamically adjusts parameter combinations to prevent convergence to local optima. Figure 6 presents the detailed optimization process.

The parameter constraints are shown as follows:

(15) $$\left\{\begin{array}{l} 70\leq m_{\mathrm{wf}}\leq 106\;\left(\mathrm{kg}\cdot s^{-1}\right)\\ 2\leq \pi \leq 7\\ 0.05\leq \psi_{R}\leq 0.5\\ 0.1\leq \psi_{H}\leq 0.6\\ 0.05\leq \psi_{L2}\leq 0.35 \end{array}\right.$$

The performance optimization of the regenerated S-CO_2_ Brayton cycle cogeneration includes five optimization variables, including $m_{\mathrm{wf}},$ π, $\psi_{R}$, $\psi_{H}$, $\psi_{L1}$. The decision to employ a neural network, as opposed to established evolutionary algorithms like NSGA-II, was primarily driven by the need to overcome computational challenges in this thermodynamic model. The traditional approach, which relies on iteratively solving a system of non-linear equations using functions like @fsolve, is computationally intensive and exhibits a strong dependence on initial guess values, often leading to convergence failures. Furthermore, frequent program interruptions caused by errors when calling the REFPROP physical property database added considerable difficulty to the optimization process.

To address these issues, we introduced a neural network as a surrogate model. Table 5 lists the parameter settings of the neural network model for the regenerated S-CO_2_ Brayton cycle cogeneration. The core advantage of this approach is that once the neural network is trained, it can instantaneously predict the system’s performance (e.g., the exergy output rate) for any given set of input parameters, bypassing the need for repeated, costly numerical solutions. This dramatically increases computational speed and eliminates the dependency on initial values.

**Figure 7:**
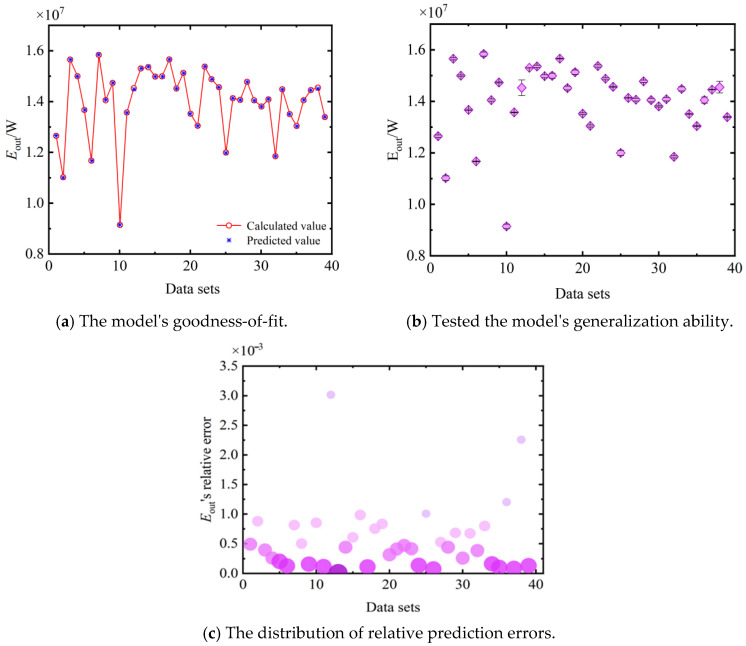
Predicted and actual calculated values of $E_{\mathrm{out}}$’s neural network.

Figure 7 presents a comparison between the predicted exergy output rate ($E_{\mathrm{out}}$) from the NN model and the actual calculated values, serving to validate the accuracy and reliability of the constructed surrogate model. Figure 7a,b show scatter plots of predicted versus actual values for the training and test sets, respectively. Figure 7c further illustrates the distribution of relative prediction errors. Overall, these results robustly demonstrate that the neural network model can serve as a high-fidelity substitute for the complex physical model in computational analyses.

The method of using the neural network is relatively simple. As long as the corresponding optimization variables are input into the neural network, the corresponding performance target can be obtained. A neural network can be thought of as a very convenient function. In the optimization process, it is only necessary to specify the value range of the corresponding optimization variables and then use the global search algorithm @globalsearch to call the neural network to obtain the optimal exergy output rate function under different conditions.

Table 6 and Table 7 present the optimization results based on the design points and selected calculation results with $E_{\mathrm{out}}$ as the optimization objective, respectively. Table 6 presents the results of the primary, secondary, and tertiary optimization of the exergy output rate $E_{\mathrm{out}}$ for the combined heat and power system featuring a recuperated S-CO_2_ Brayton cycle coupled with a series-connected water heater. Table 6 shows the results of the third-stage optimization with $E_{\mathrm{out}}$ as the objective function. The data indicate that $E_{\mathrm{out}}$ initially increases and then decreases with increasing mass flow rate, while the corresponding optimal pressure ratio gradually decreases. Concurrently, $\psi_{R}$ and $\psi_{H}$ exhibit increasing trends, whereas $\psi_{L2}$ and $\psi_{L1}$ demonstrate decreasing trends. The increase in $\psi_{R}$ enhances internal heat recovery, reducing the exergy destruction associated with the large temperature difference in the heater and lowering the system’s dependency on the high-temperature heat source. The optimized increase in $\psi_{L1}$ leads to a lower compressor inlet temperature, which directly reduces the compression work and contributes to a higher net power output. The decrease in $\psi_{H}$ indicates that the system’s performance is more significantly boosted by optimizing the internal heat recovery and compression process than by maximizing heat addition from the external source under a fixed total conductance constraint.

The first optimization stage increases $E_{\mathrm{out}}$ by 5.43%, mainly due to the redistribution of conductance from the heater to the recuperator and cooler. The second stage yields a further 11.6% gain by adjusting $m_{\mathrm{wf}}$ to 83.94 kg/s, demonstrating that mass flow strongly influences performance. Finally, releasing the pressure ratio variable achieves a global optimum at π = 5.64 and $m_{\mathrm{wf}}$ = 79 kg/s, with $E_{\mathrm{out}}$ improving by 16.06% relative to the baseline. These results confirm that the combined effect of conductance allocation, reduced flow, and moderate pressure ratio maximizes cycle performance. The 16.06% improvement achieved here is comparable to or higher than improvements reported for other recuperated S-CO_2_ cycle optimizations [32,34,35,36], indicating that exergy-based multi-parameter optimization provides stronger performance gains than single-variable adjustments.

### 3.4. Exergy Destruction Analysis

This section presents an exergy destruction analysis based on the exergy balance principle and the Gouy–Stodola theorem [51].

**Figure 8:**
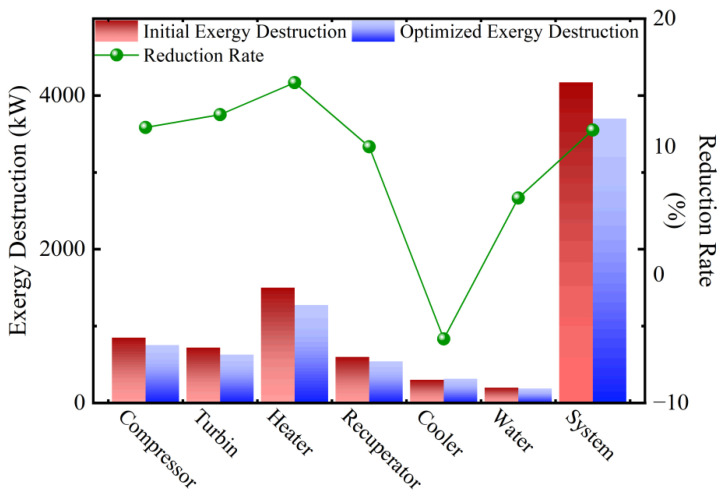
Comparisons of exergy destructions and corresponding reduction rates for different components.

As shown in Table 8 and Figure 8, the system optimization process successfully reduced the total exergy destruction by 11.3%. This demonstrates that the optimization targeting the maximization of exergy output rate not only enhanced the system’s useful output but also effectively suppressed internal irreversibilities through improved parameter matching and thermal conductance allocation. The most significant improvement was observed in the heater, where exergy destruction was reduced by 15.0%. However, the exergy destruction of the cooler increased by 5.0% after optimization. The fundamental reason for this is that to maximize the overall system performance, the thermal conductance allocation to the cooler was increased (*ψ*_L1_ from 0.300 to 0.345). Although this slightly intensified the irreversibility of this specific component, it yielded greater system-level benefits by lowering the compressor inlet temperature.

## 4. Conclusions

This paper establishes a finite time thermodynamics (FTT) model for a series-connected combined heat and power system integrating a recuperated supercritical carbon dioxide (S-CO_2_) Brayton cycle with a water heater. By incorporating a neural network with decision variables including the working fluid mass flow rate, cycle pressure ratio, and thermal conductance allocation ratios of the heat exchangers, optimization was conducted with the maximization of exergy output rate as the objective. This study demonstrates the following:

(1) For fixed compressor and turbine efficiencies, total thermal conductance, and working fluid mass flow rate, there exists an optimal pressure ratio that maximizes the exergy output rate. As the compressor and turbine efficiencies increase, the irreversibility within the system decreases, leading to an increase in both the exergy output rate and its corresponding optimal pressure ratio.
(2) With the objective of maximizing the exergy output rate $E_{\mathrm{out}}$, performance analysis and optimization of the cycle were carried out. Under varying conditions of working fluid mass flow rate $m_{\mathrm{wf}}$, turbine efficiency $\eta_{t}$, compressor efficiency $\eta_{c}$, and thermal conductance allocation ratio of the water heater $\psi_{L2}$, an optimal pressure ratio exists that maximizes the cycle exergy output rate. The optimal parameter combination was found to be a working fluid mass flow rate of 79 $\mathrm{kg}\cdot s^{-1}$ and a pressure ratio of 5.64. After optimization, the system’s exergy output rate improved by 16.06%. Increasing the thermal conductance allocation ratios of the recuperator and the cooler, while decreasing that of the heater, can enhance the system exergy output rate. The obtained results could provide theoretical guidance for the design of S-CO_2_ Brayton cycle cogeneration systems, with potential applications in industrial waste heat recovery and marine power systems.
(3) This study has limitations, such as the assumption of constant total thermal conductance and fixed component efficiencies. Future work could consider part-load conditions, multi-objective optimization, and the integration of renewable energy sources.

## Figures and Tables

**Table 1:** Initial values of selected parameters are calculated for model validation [50].

Parameter	Value	Unit
$m_{\mathrm{wf}}$	12.65	$\mathrm{kg}\cdot s^{-1}$
$p_{\max}$	19.31	MPa
$p_{\min}$	7.63	MPa
$\eta_{t}$	0.8	——
$\eta_{c}$	0.8	——
$m_{H}$	9	$\mathrm{kg}\cdot s^{-1}$
$m_{L}$	50	$\mathrm{kg}\cdot s^{-1}$
$T_{H,\mathrm{in}}$	792.15	K
$T_{L,\mathrm{in}}$	298.15	K

**Table 2:** Comparison of model validation results.

Parameter	Reference [50]	This Paper	Comparative Results
*T*_1_	305.15 K	305.44 K	0.01%
*T*_2_	335.88 K	347.88 K	3.57%
*T*_3_	485.25 K	480.85 K	−0.91%
*T*_4_	673.15 K	680.23 K	1.05%
*T*_5_	584.02 K	598.31 K	2.45%
*T*_6_	344.97 K	347.37 K	0.70%
*W*_net_	0.80 MW	0.82 MW	2.50%

**Table 3:** Comparison of model validation results ($\eta_{t}$ = 0.8, $\eta_{c}$ = 0.85).

Parameter	Reference [50]	This Paper	Comparative Results
*T*_1_	305.15 K	305.45 K	0.01%
*T*_2_	335.88 K	339.80 K	3.92%
*T*_3_	485.25 K	485.01 K	−0.05%
*T*_4_	673.15 K	680.15 K	1.04%
*T*_5_	584.02 K	582.99 K	−0.02%
*T*_6_	344.97 K	335.20 K	2.83%
*W*_net_	0.80 MW	0.79 MW	−1.25%

**Table 4:** Initial design parameters of the cogeneration plant.

Parameter	Value	Unit
$T_{H,\mathrm{in}}$	805.15	K
$T_{L1,\mathrm{in}}$	298.15	K
$T_{L2,\mathrm{in}}$	298.15	K
$m_{H}$	89.9	$\mathrm{kg}\cdot s^{-1}$
$m_{L1}$	1000	$\mathrm{kg}\cdot s^{-1}$
$m_{L2}$	100	$\mathrm{kg}\cdot s^{-1}$
$m_{\mathrm{wf}}$	100	$\mathrm{kg}\cdot s^{-1}$
$p_{\min}$	7.7	MPa
$p_{\max}$	30.8	MPa
$\eta_{c}$	0.89	——
$\eta_{t}$	0.89	——
$\psi_{H}$	0.45	——
$\psi_{R}$	0.15	——
$\psi_{L1}$	0.3	——
$\psi_{L2}$	0.1	——
$c_{p,L}$	4181.3	$\mathrm{kJ}/\left(\mathrm{kg}\cdot K\right)$
$c_{p,H}$	1103.7	$\mathrm{kJ}/\left(\mathrm{kg}\cdot K\right)$
*UA*_T_	3000	kW/K

**Table 5:** The parameter settings of the neural network model for the regenerated S-CO_2_ Brayton cycle cogeneration.

Parameter Name	$E_{\mathrm{out}}$	*T*_1_
samples	8000	8000
input nodes	4	4
output	1	1
Hidden layers	2	2
Number of hidden layer nodes layer nodes	30, 15	30, 15
Hidden layer activation function	tansig, logsig	tansig, logsig
Training times	80,000	80,000
Minimum number of confirmation failures	60	39
Learning rate	1.0	0.4
Minimum training target error	3 × 10^−6^	2 × 10^−7^
Performance function	mse	mse

**Table 6:** $E_{\mathrm{out}}$ optimization calculation results based on design points.

Parameters and Objective	Initial Design Point	First Optimization Result	Second Optimization Result	Third Optimization Result
π	6.570	6.570	2.950	5.640
$m_{\mathrm{wf}}/\mathrm{kg}\cdot s^{-1}$	100.00	100.00	83.94	79.00
$\psi_{R}$	0.150	0.185	0.229	0.161
$\psi_{H}$	0.450	0.382	0.346	0.301
$\psi_{L1}$	0.300	0.264	0.285	0.345
$\psi_{L2}$	0.100	0.164	0.184	0.192
$E_{\mathrm{out}}/\times 10^{6}\;W$	15.093	15.912	16.844	17.517
$\delta E_{\mathrm{out}}/\%$	——	5.426	11.601	16.062

**Table 7:** Calculation results with $E_{\mathrm{out}}$ as the optimization objective.

Optimization Variables						Optimization Objective	Optimization Results
$m_{\mathrm{wf}}/\mathrm{kg}\cdot s^{-1}$	π	$\psi_{R}$	$\psi_{H}$	$\psi_{L1}$	$\psi_{L2}$	*E*_out_/×10^6^ W	*δE*_out_/%
70	6.744	0.050	0.200	0.406	0.344	17.297	14.603
73	6.200	0.090	0.230	0.386	0.293	17.318	14.741
76	6.021	0.111	0.251	0.355	0.283	17.484	15.842
79	5.640	0.161	0.301	0.345	0.192	17.517	16.062
82	5.201	0.192	0.321	0.314	0.172	17.457	15.666
85	5.354	0.222	0.352	0.304	0.122	17.416	15.393
88	4.601	0.243	0.382	0.263	0.112	17.103	13.317
91	4.202	0.273	0.392	0.232	0.103	16.681	10.522
94	4.101	0.303	0.403	0.173	0.121	16.372	8.472
97	3.843	0.344	0.429	0.141	0.086	15.436	4.921
100	3.203	0.395	0.443	0.100	0.063	14.504	3.387
103	2.994	0.403	0.445	0.100	0.052	14.304	2.235
106	2.544	0.411	0.455	0.100	0.034	14.283	1.371

**Table 8:** Exergy destruction analysis of major system components.

Component	Initial Exergy Destruction (kW)	Optimized Exergy Destruction (kW)	Reduction Rate (%)
Compressor	850	752	11.5
Turbin	720	630	12.5
Heater	1500	1275	15.0
Recuperator	600	540	10.0
Cooler	300	315	−5.0
Water Heater	200	188	6.0
System Total	4170	3700	11.3

## Data Availability

The original contributions presented in this study are included in the article. Further inquiries can be directed to the corresponding author.

## Nomenclature

*c_p_*	Specific heat capacity at constant pressure, J·kg^−1^·K^−1^
*E*	Ecological function, W
*h*	Specific enthalpy, J·kg^−1^
*m*	Mass flow rate, kg·s^−1^
*P*	Power, W
*P*	Pressure, Mpa
*Q*	Heat transfer rate, W
*T*	Temperature, K
*U*	Heat conductance, $\mathrm{kW}\cdot K^{-1}$
W	Net power output, W
**Greek letters**	
ψ	Heat conductance distribution ratio
π	Pressure ratio
δ	Lowercase delta
*η*	Efficiency
**Subscripts**	
c	Compressor
H	Heat source
hg	Flue gas
K	User
in	Inlet or inside
L	Cold source
Max	Maximum
Min	Minimum
out	Outlet or outside
R	Regenerator
T	Gross amount
t	Turbine
wf	Working fluid
0	Ambient temperature
1–7	State points
**Abbreviations**	
CCES-CCHP	Comprehensive combined energy system-combined cooling, heating and power
CCHP	Combined cooling, heating, and power
CHP	Combined heat and power
CSP	Concentrated solar power
EGM	Entropy Generation Minimization
FTT	Finite-time thermodynamics
NN	Neural network
NSGA-II	Non-dominated sorting genetic algorithm II
TC-CCES	Transcritical compressed carbon dioxide energy storage system
S-CO_2_	Supercritical Carbon-dioxide
OO	Optimization objective
OV	Optimization variable
SOO	Single-objective optimization
